# A Meta-Analysis of Efficacy and Safety of PD-1/PD-L1 Inhibitors in Triple-Negative Breast Cancer

**DOI:** 10.1155/2022/2407211

**Published:** 2022-01-21

**Authors:** Chaoyang Wang

**Affiliations:** Department of Oncological Surgery, Hanzhong 3201 Hospital Affiliated to Xi'an Jiaotong University, Hanzhong, China

## Abstract

**Objective:**

To evaluate the efficacy and safety of programmed cell death receptor-1 (PD-1)/programmed cell death-ligand protein-1 (PD-L1) inhibitors in triple-negative breast cancer (TNBC) to provide a treatment basis for TNBC.

**Methods:**

Published case-control studies on PD-1/PD-L1 inhibitors in the treatment of TNBC were retrieved from PubMed, Embase, and Cochrane Library databases, and collected data were processed by RevMan 5.4.

**Results:**

A total of 7 studies with 4340 study subjects were obtained, including 2092 PD-L1-negative cases, 1375 PD-L1-positive cases, and 847 PD-L1 unidentified cases. The use of PD-1/PD-L1 inhibitors showed no significant impact on patients' progression-free survival (PFS) and overall survival (OS). The use of PD-1/PD-L1 inhibitors in the PD-L1-positive subgroup significantly improved patients' PFS and OS. Treatment with PD-1/PD-L1 inhibitors presented no significant effect on the incidence of adverse events (AEs) but increased the risk of AE grade ≥3 and severe AEs (SAEs).

**Conclusion:**

PD-1/PD-L1 inhibitors are effective in the treatment of TNBC, which is strongly correlated with the expression of PD-L1; patient selection and clinical application require further investigation and verification.

## 1. Introduction

Breast cancer is the uncontrolled proliferation of breast epithelial cells induced by multiple oncogenic factors [1]. Its early manifestations include breast lumps, nipple overflow, and enlarged axillary lymph nodes, and distant metastasis of the disease in the advanced stage may develop multiorgan lesions, which is life-threatening [2]. The molecular types of breast cancer include Ki-67, estrogen receptor (ER), progesterone receptor (PR), and human epidermal growth factor receptor-2 (HER2), which provide diagnostic and prognostic evidence and guidance for treatment [3]. Triple-negative breast cancer (TNBC) is a breast cancer with the inexpression of ER, PR, and HER2, which accounts for 12% of all breast cancer cases and is characterized by a young age of onset, high invasiveness, and easy recurrence and metastasis [4]. The 5-year survival of early-stage breast cancer exceeds 90%, that of patients with early-stage TNBC decreases to 77%, and that of patients with advanced TNBC remains only 14% [5]. The current treatments for TNBC are severely circumscribed owing to the insufficiency of effective therapeutic targets. The main treatment for advanced TNBC relies on chemotherapy with traditional anthracyclines, paclitaxel, and platinum [6] to prolong patients' survival, but its efficiency still leaves much to be desired. Besides, intensive chemotherapy may also seriously compromise patients' quality of life [7].

Programmed death protein-1 (PD-1) and its ligand (PD-L1) inhibitors are immune sentinel monoclonal antibodies with outstanding action range, depth, and durability of response. Immunotherapy with PD-1/PD-L1 inhibitors is a new and highly appreciated anticancer immunotherapy that reactivates the body's immune system to defend against cancer cells by blocking the PD-1/PD-L1 signaling pathway [8, 9]. Currently, the marketed PD-1 inhibitors including nivolumab, pembrolizumab, and tislelizumab are mainly used for the treatment of melanoma and non-small-cell lung cancer, and their efficacy against renal cell carcinoma, bladder cancer, and Hodgkin's lymphoma is still under large-scale clinical trials [10, 11]. Atezolizumab, durvalumab, and avelumab are PD-L1 inhibitors that have been clinically approved for the treatment of urothelial carcinoma, and many other drugs are currently undergoing early clinical trials [12, 13]. Currently, targeted PD-1/PD-L1 inhibitors have been ratified for the treatment of hepatocellular carcinoma, renal cancer, and advanced non-small-cell lung cancer, and atezolizumab is the first PD-L1 antibody ratified for advanced TNBC. PD-1/PD-L1 inhibitors combined with chemotherapy have been reported to show great potential as a new TNBC therapy [14]. However, the outcome assessment of TNBC patients treated with PD-1/PD-L1 inhibitors still warrants further confirmation. Accordingly, this study was to evaluate the clinical efficacy of patients with TNBC receiving PD-1/PD-L1 inhibitors by meta-analysis to provide guidance for the application of PD-1/PD-L1 inhibitors in patients with advanced TNBC.

## 2. Materials and Methods

### 2.1. Literature Retrieval

A literature search in PubMed, Embase, and Cochrane Library was conducted from database establishment to August 30, 2021, in the language of both English and Chinese, with the search terms of (“PD-1” or “PD-L1” or “PD-1/PD-L1” or “programmed cell death 1” or “programmed cell death-ligand 1”) and (“breast cancer” or “mammary cancer” or “Breast Carcinoma” or “Breast Tumor” or “TNBC”) and their corresponding Chinese terms. The search was conducted twice to avoid omission.

### 2.2. Literature Inclusion and Exclusion Criteria

#### 2.2.1. Inclusion Criteria

The inclusion criteria were as follows: (1) study type: randomized controlled clinical trial; (2) study subjects: pathologically diagnosed breast cancer with clear ER, PR, and HER2 negative confirmed by immunohistochemistry (IHC) and fluorescence in situ hybridization (FISH); (3) interventions: after randomization, at least two groups were divided, one of which received immune-targeted therapy combined with radiotherapy; (4) study indicators: one or more of overall survival (OS), progression-free survival (PFS), and adverse events (AEs); (5) scientific and standardized study design, with clear grouping and interventions, and well-documented follow-up data.

#### 2.2.2. Exclusion Criteria

The exclusion criteria were as follows: (1) nonrandomized controlled clinical trials or nonprimary studies; (2) unavailability of relevant outcome indicators such as OS, PFS, and AEs; (3) inclusion of fewer than 20 patients.

#### 2.2.3. Screening of the Included Literature

The data were searched by two researchers, and literature management was performed using Endnote. Duplicate literature was excluded, and initial screening of the search results was carried out with regard to the titles, abstracts, and full text, followed by the inclusion of the literature according to the inclusion criteria. The Newcastle-Ottawa Scale was used to evaluate the quality of the included literature, and the decision for inclusion was independently assessed by a third investigator in case of disagreement between the two investigators.

### 2.3. Data Extraction

Data extraction and collation were performed independently by two investigators, including authors, time of publication, subject type, intervention method, use or absence of blinding, and randomization. OS, PFS, and AEs were considered the main effect measures for meta-analysis.

### 2.4. Risk of Bias

The risk of bias of the included literature was assessed in terms of the following six dimensions: random sequence generation (selection bias), allocation concealment (selection bias), blinding of participants and personnel (performance bias), blinding of outcome assessment (detection bias), incomplete outcome data (attrition bias), and selective reporting (reporting bias), and was categorized into three types: low risk, high risk, and uncertain risk.

### 2.5. Statistical Analyses

RevMan 5.4 was used to organize and analyze the data, and the generic inverse variance was selected for the hazard ratio (HR) data to compute and record the log [HR] and the corresponding SE. Dichotomous data types were selected for data such as adverse events. The heterogeneity of the included literature was evaluated by the Cochrane *Q* test and *I*^2^ test. *I*^2^ = 0 and *P* > 0.1 in both subgroups indicate no heterogeneity in the included studies whose analyses were subjected to a fixed-effect model. *I*^2^ > 0 and *P* < 0.1 in both subgroups indicate heterogeneity in the included studies whose analyses were subject to a random-effect model. Egger's and Begg's tests were used to assess publication bias.

## 3. Results

**Figure 1:**
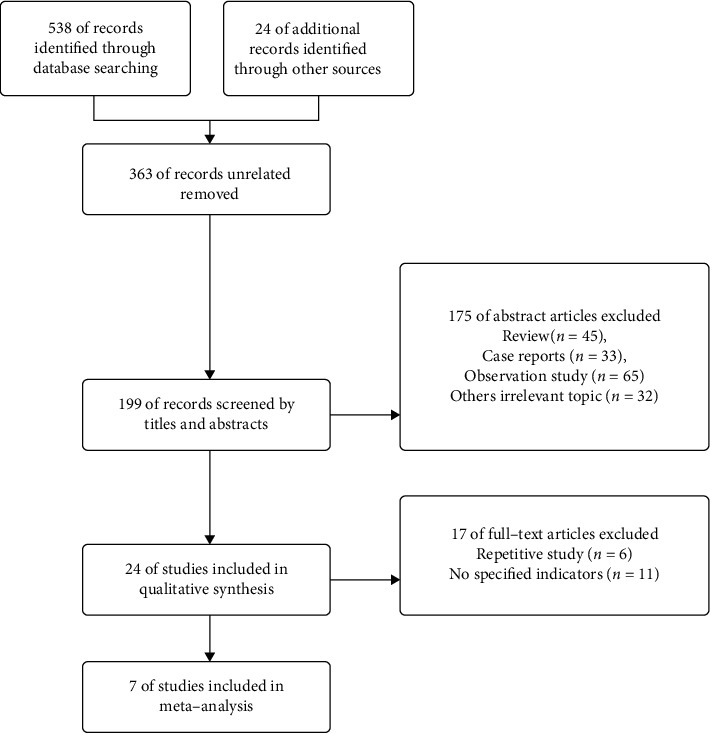
Study flow diagram.

Of 1538 original papers retrieved, 1491 papers were excluded, and 47 were preliminarily included after reviewing the abstracts and excluding duplicate papers, abstracts, and reviews. After reading the full texts, studies with issues such as duplicate reports and no specified data were excluded, and seven papers were finally included. The study flow diagram is shown in Figure 1, and the basic information of the literature is shown in Table 1.

**Figure 2:**
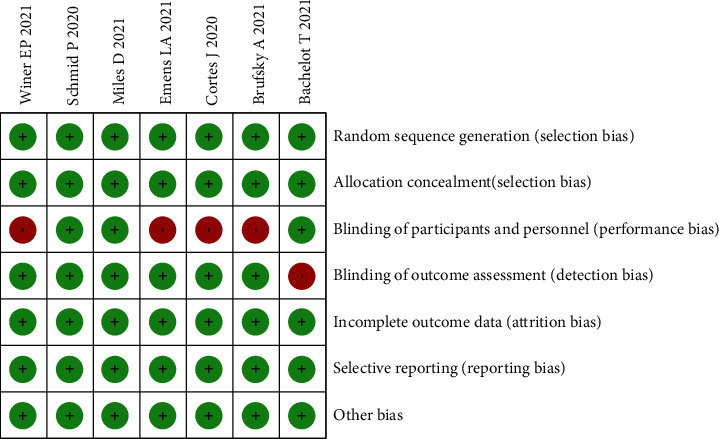
Risk of bias summary: review for the enrolled study.

The results of the Cochrane risk bias assessment are shown in Figure 2. All seven included studies were of high quality and mostly at low risk of bias (green section in the figure). Four studies had performance bias, and one study had detection bias due to the failure of the study design to meet double-blind requirements for informed consent (red section in the figure).

**Figure 3:**
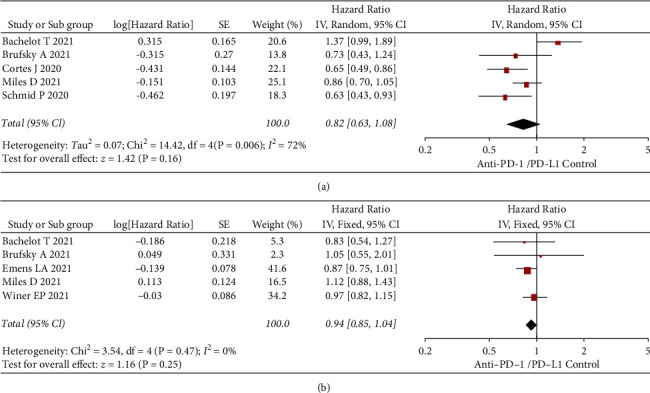
Forest plots of anti-PD-1/PD-L1 in overall TNBC. (a) PFS. (b) OS. SE: standard error; CI: confidence interval; TNBC: triple-negative breast cancer; PD: progression death; OS: overall survival; PFS: progression-free survival.

Of the 7 included studies, 5 included all-subject PFS outcomes, and 5 included all-subject OS outcomes. As shown in Figure 3(a), in the PFS analysis of all subjects, there was significant within-group heterogeneity (*I*^2^ = 72% and *P*=0.006), and the HR result was (0.82 [0.63, 1.08], *Z* = 1.42, *P*=0.16) using the random-effect model. As shown in Figure 3(b), there was no significant within-group heterogeneity in the OS analysis (*I*^2^ = 0 and *P*=0.47), and the HR result was (0.94 [0.85, 1.04], *Z* = 1.16, *P*=0.25). The results of the meta-analysis in all subjects showed that the use or absence of PD-1/PD-L1 inhibitors had no significant effect on patients' PFS and OS (all *P* > 0.05).

**Figure 4:**
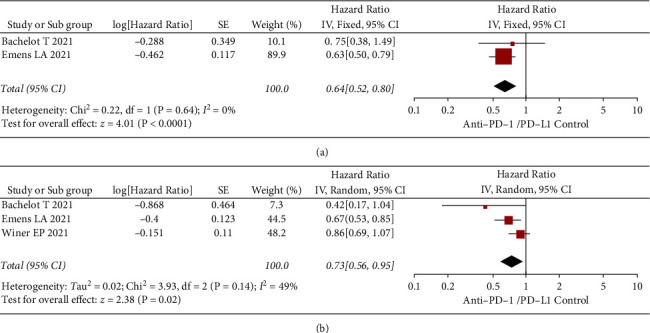
Forest plots of anti-PD-1/PD-L1 in the PD-L1-positive subgroup TNBC. (a) PFS. (b) OS. SE: standard error; CI: confidence interval; TNBC: triple-negative breast cancer; PD: progression death; OS: overall survival; PFS: progression-free survival.

The subjects were divided into PD-L1 negative and PD-L1 positive according to PD-L1 expression, with two studies containing the PFS results for the PD-L1-positive subgroup and three studies containing the OS results for the PD-L1-positive subgroup. As shown in Figure 4(a), there was no significant within-group heterogeneity in the PFS analysis of the PD-L1-positive subgroup (*I*^2^ = 0 and *P*=0.64), and the HR result was (0.64 [0.52, 0.80], *Z* = 4.01, *P* < 0.0001) using a fixed-effect model. As shown in Figure 4(b), there was significant within-group heterogeneity in the OS analysis in the PD-L1-positive subgroup (*I*^2^ = 49% and *P*=0.14), and the HR result was (0.73 [0.56, 0.95], *Z* = 2.38, *P*=0.02) using a random-effect model. The results of the meta-analysis in the PD-L1-positive subgroup showed that the use of PD-1/PD-L1 inhibitors significantly improved PFS and OS in patients (all *P* < 0.05).

**Figure 5:**
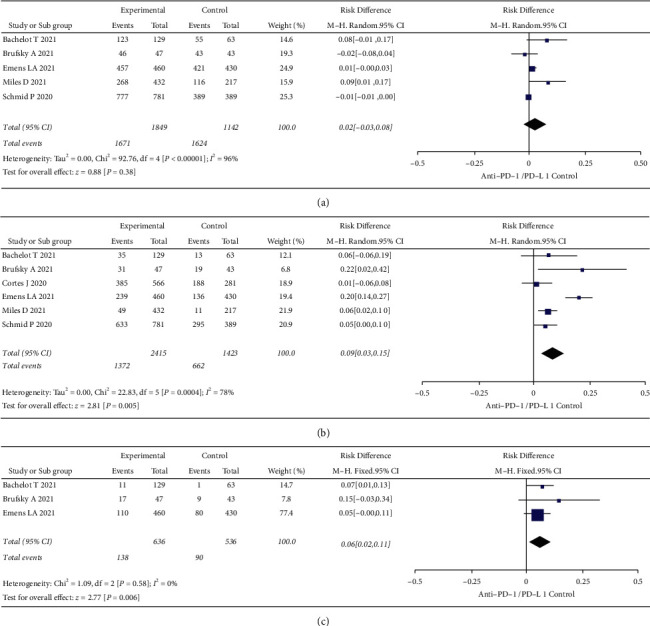
Forest plots of AEs. (a) All-grade AEs. (b) Grade ≥3 AEs. (c) SAEs. SE: standard error; CI: confidence interval; TNBC: triple-negative breast cancer; PD: progression death; AEs: adverse events; SAEs: severe adverse events.

Six studies included all AE outcomes during treatment, three of which AEs were classified as all grade, grade ≥3, and SAE, two as all grade and grade ≥3, and one as grade ≥3. As shown in Figure 5(a), there was significant within-group heterogeneity (*I*^2^ = 96% and *P* < 0.0001) in the analysis of subjects with all-grade AEs, and the risk difference was (0.02 [−0.03, 0.80], *Z* = 0.88, *P*=0.38) using the random-effect model. As shown in Figure 5(b), there was significant within-group heterogeneity in the analysis of subjects with grade ≥3 AEs (*I*^2^ = 78% and *P*=0.0004), and the risk difference results were (0.09 [0.03, 0.15], *Z* = 2.81, *P*=0.005) using the random-effect model. As shown in Figure 5(c), there was no significant within-group heterogeneity in the analysis of all-grade SAEs in subjects (*I*^2^ = 0 and *P*=0.58), and the risk difference result was (0.06 [0.02, 0.11], *Z* = 2.77, *P*=0.006) using the fixed-effect model. Treatment with PD-1/PD-L1 inhibitors showed no significant effect on all-grade AEs (*P* > 0.05), but increased the risk of grade ≥3 AEs and SAEs (all *P* < 0.05).

## 4. Discussion

Currently, chemotherapy remains the basic treatment for TNBC. Notwithstanding the progress of chemotherapy combined with antiangiogenic drugs and immune-targeted drugs, the efficacy is still unsatisfactory. Research has revealed higher levels of PD-L1 in the tumor microenvironment of TNBC compared to other breast cancers, for which immunosuppressive therapy targeting PD-1/PD-L1 may provide a novel therapeutic insight [22]. T lymphocytes recognize and kill tumor cells. PD-1 is a T-lymphocyte regulatory protein that reduces the tumor-killing effect of T lymphocytes, which may contribute to tumorigenesis. PD-L1 is a ligand protein produced by tumor cells that binds PD-1 to attenuate T-cell-mediated immunosurveillance and provide an immune escape from cancer cells [23]. PD-1/PD-L1 inhibitors can specifically bind PD-1/PD-L1 and restore the normal immune function of T lymphocytes to kill tumor cells. In recent years, the application of PD-1/PD-L1 inhibitors in TNBC has achieved considerable progress, but the treatment efficiency remains unfavorable and circumscribed.

In this study, a meta-analysis was performed on seven RCT studies in which four drugs were employed, including atezolizumab used in two studies [18, 19], durvalumab in one [15], cobimetinib in one [16], and pembrolizumab in three [17, 20, 21]. Atezolizumab was the first PD-L1 inhibitor approved by the FDA and the first immunotherapy regimen for breast cancer. In the IMpassion130 study [18], first-line treatment of TNBC with atezolizumab in combination with paclitaxel achieved favorable PFS results in all patients, with a more pronounced advantage in the PD-L1-positive patient group, but the difference in OS between the two groups was not statistically different. However, the advantages of combining atezolizumab with paclitaxel for the treatment of metastatic TNBC were absent from the results of IMpassion131 [19] compared to the previously published trial results of the IMpassion130 study, and the reasons for the absence of differences remain elusive. Durvalumab is a PD-L1 antibody with promising efficacy in bladder and lung cancers. In the SAFIR02-BREAST IMMUNO study [15], durvalumab improved OS in TNBC patients but showed no significant difference in the subgroup analyses. Furthermore, an exploratory analysis of TNBC patients showed an HR of 0.18 for durvalumab efficacy in patients with CD274 gene gain/amplification and 1.12 in patients with normal/lost CD274 gene, which provides a new reference for the selection of the durvalumab treatment population.

The results of this study showed no significant effect of PD-1/PD-L1 inhibitors on PFS and OS in patients without PD-L1 expression classification but significantly improved the PFS and OS of patients in the PD-L1-positive subgroup, which confirms the close correlation between the effect of PD-1/PD-L1 inhibitors and PD-L1 expression. Differences between this result and the results of separate studies may be attributed to the following reasons. The first is the difference in the determination of PD-L1 expression. PD-L1 expression is determined through immunohistochemistry (IHC) and is found on both tumor cells and immune cells. However, PD-L1 is a versatile but not yet impeccable biomarker for predicting anti-PD-1 or anti-PD-L1 antibodies in patients with various tumors [24]. The second is the difference in the method of drug administration, whose application methods include combination chemotherapy and preoperative neoadjuvant chemotherapy, with certain variations in efficacy. In addition, a meta-analysis of AEs in this study showed that PD-1/PD-L1 inhibitors increase the risk of high-grade AEs and SAEs. Nonetheless, the source of adverse events, from medication issues or the expression of PD-L1, remains unconfirmed as the type of intervention is determined in the subjects based on PD-L1 expression levels.

In conclusion, PD-1/PD-L1 inhibitors are effective in the treatment of TNBC, which is strongly correlated with the expression of PD-L1, and patient selection and clinical application require further investigation and verification.

## Figures and Tables

**Table 1:** Basic information of the enrolled literature.

Author	Year	Phase	Patient	Arm 1	Arm 2	Patients			
						Total	PD-L1 negative	PD-L1 positive	Outcomes
Bachelot et al. [15]	2021	II	TNBC	Durvalumab	Maintenance chemotherapy	82	32	29	OS, PFS, AEs
Brufsky et al. [16]	2021	II	mTNBC	Cobimetinib	Placebo + paclitaxel	62	44	18	OS, PFS, AEs
Cortes et al. [17]	2020	III	mTNBC	Pembrolizumab	Placebo + chemotherapy	847	—	—	PFS, AEs
Emens et al. [18]	2021	III	TNBC	Atezolizumab	Placebo + nab-paclitaxel	902	533	369	OS, PFS, AEs
Miles et al.[19]	2021	III	mTNBC	Atezolizumab	Placebo + paclitaxel	651	293	358	PFS, OS, AEs
Schmid et al. [20]	2020	III	Stage II/III TNBC	Pembrolizumab	Placebo	1174	973	196	PFS, AEs
Winer et al. [21]	2021	III	mTNBC	Pembrolizumab	Chemotherapy	622	217	405	OS, PFS, AEs

TNBC: triple-negative breast cancer; PD: progression death; OS: overall survival; PFS: progression-free survival; AEs: adverse events.

## Data Availability

The datasets used during the present study are available upon request to the author.
